# Proteomic Study of Broiler Plasma Supplemented with Different Levels of Copper and Manganese from Different Sources

**DOI:** 10.3390/molecules28248155

**Published:** 2023-12-18

**Authors:** Renata Aparecida Martins, Andrey Sávio de Almeida Assunção, José Cavalcante Souza Vieira, Leone Campos Rocha, Priscila Michelin Groff Urayama, Marília Afonso Rabelo Buzalaf, José Roberto Sartori, Pedro de Magalhães Padilha

**Affiliations:** 1 School of Veterinary Medicine and Animal Science, São Paulo State University (UNESP), Botucatu 18618-681, SP, Brazil; renata.a.martins@unesp.br (R.A.M.); asa.assuncao@unesp.br (A.S.d.A.A.); leone.rocha@unesp.br (L.C.R.); priscila.groff@unesp.br (P.M.G.U.); jose.sartori@unesp.br (J.R.S.); 2 Institute of Biosciences, São Paulo State University (UNESP), Botucatu 18618-693, SP, Brazil; cavalcante.vieira@unesp.br; 3 Bauru School of Dentistry, University of São Paulo (USP), Bauru 17012-901, SP, Brazil; mbuzalaf@fob.usp.br

**Keywords:** minerals supplementation sources, copper and manganese bioavailability, broiler nutrition, proteomics, metalloproteomics

## Abstract

The aim of the present study was to evaluate the differential expression of plasma proteins in broiler chickens supplemented with different sources (sulfates and hydroxychlorides) and levels of copper (15 and 150 mg kg^−1^) and manganese (80 and 120 mg kg^−1^). For this, plasma samples from 40 broiler chickens were used, divided into four experimental groups: S15-80 (15 ppm CuSO_4_ and 80 ppm MnSO_4_), S150-120 (150 ppm CuSO_4_ and 120 ppm MnSO_4_), H15-80 (15 ppm Cu(OH)Cl and 80 ppm Mn(OH)Cl), and H150-120 (150 ppm Cu(OH)Cl and 120 ppm Mn(OH)Cl). From plasma samples obtained from each bird from the same treatment, four pools were made considering 10 birds per group. Plasma proteome fractionation was performed by 2D-PAGE. Concentrations of the studied minerals were also evaluated in both plasma and protein pellet samples. A higher concentration of Cu and Mn was observed in the plasma and protein pellets of groups that received higher mineral supplementation levels compared to those receiving lower levels. Mn concentrations were higher in plasma and protein pellets of the hydroxychloride-supplemented groups than the sulfate-supplemented groups. Analysis of the gels revealed a total of 40 differentially expressed spots among the four treatments. Supplementation with different sources of minerals, particularly at higher levels, resulted in changes in protein regulation, suggesting a potential imbalance in homeostasis.

## 1. Introduction

In poultry, micromineral supplementation is essential for the normal development and health of birds since diets based on corn and soybean meal do not have sufficient concentrations to meet nutritional requirements [1,2]. Among the most studied minerals in broiler nutrition are copper and manganese, as they play several important roles in metabolism and directly impact productivity in situations of deficiency [1,3,4]. For example, both copper and manganese are essential in antioxidant defense by acting as catalytic cofactors of superoxide dismutase (CuZn-SOD and Mn-SOD), in addition to many other metalloproteins involved in various vital biochemical functions for animals and humans [1,4,5,6].

Copper and manganese are usually supplemented from inorganic sources such as sulfates, oxides and carbonates. Although these sources have a low cost and a large commercial supply, the low mineral bioavailability presented encourages supplementation in high amounts, which may exceed the recommended nutritional levels and, consequently, cause undesirable effects on the health of birds [2,6,7]. For this reason, the use of inorganic sources presents significant challenges, driving the search and development of other more efficient forms of mineral supplementation [8]. Hydroxychlorides are an alternative with promising characteristics to replace traditionally used sources. Due to their crystalline structure, hydroxychlorides can provide a slower release of minerals in the gastrointestinal tract, in addition to having lower hygroscopicity and water solubility, making them less reactive with other feed nutrients [9,10,11].

Studies were carried out to compare hydroxychlorides with other sources, as well as the effects of supplementation at different levels on parameters such as performance, carcass traits and mineral content in liver, plasma and tibial bone [12,13,14,15,16]. However, further studies are needed to evaluate the influence on the protein profile of tissues and biological fluids, considering that microminerals, especially copper and manganese, act as metallic components of many proteins. In addition, the analysis of protein expression allows for the study of the mechanisms involved in the tolerance and detoxification of metals, since the proteome responds to several stressful conditions and is extremely useful in the evaluation of high concentrations of minerals [17].

In this sense, the present research is a preliminary study in which the proteomic technique of two-dimensional electrophoresis in polyacrylamide gel (2D-PAGE) was used, with the objective of evaluating the plasmatic proteome of broiler chickens supplemented with two levels of Cu (15 and 150 mg kg^−1^) and Mn (80 and 120 mg kg^−1^) through sulfates and hydroxychlorides.

## 2. Results

### 2.1. Fractionation of Broiler Plasma Proteome

**Figure 1:**
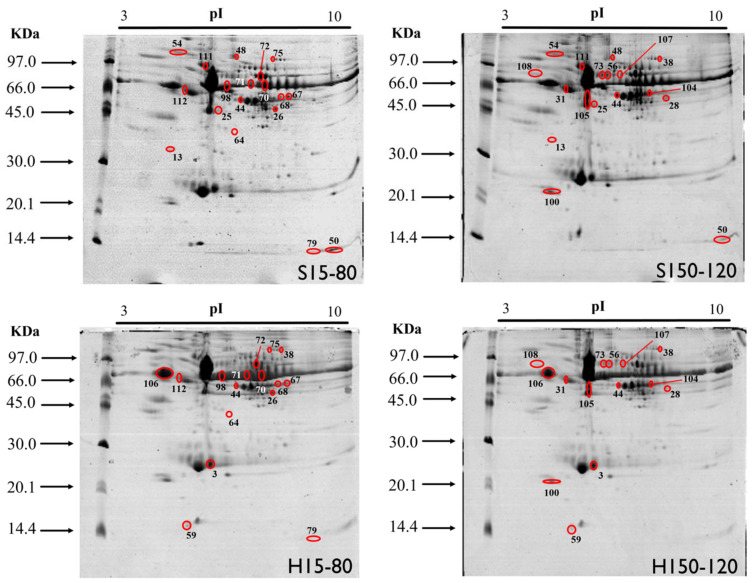
Representation of the polyacrylamide gels obtained by 2D-PAGE from the pool of plasma samples from broiler chickens supplemented with different sources and levels of Cu and Mn. The differentially expressed spots between treatments are circled in red and identified with numbers. S15-80 (15 mg kg^−1^ Cu sulfate and 80 mg kg^−1^ Mn sulfate), S150-120 (150 mg kg^−1^ Cu sulfate and 120 mg kg^−1^ Mn sulfate), H15-80 (15 mg kg^−1^ Cu hydroxychloride and 80 mg kg^−1^ Mn hydroxychloride) and H150-120 (150 mg kg^−1^ Cu hydroxychloride and 120 mg kg^−1^ Mn hydroxychloride).

The triplicates of the gels obtained showed an average number of 229 ± 17 spots and an average correspondence of 99 ± 1%, indicating good reproducibility and repeatability of the gels (Figure 1).

### 2.2. Copper and Manganese Determinations

In the samples of plasma and protein pellets, the S150-120 and H150-120 groups had the highest concentrations of Cu, while the S15-80 group had the lowest concentration (*p* < 0.05) (Table 1). There was no difference in Cu concentration in the samples and pellets between the S150-120 and H150-120 groups (Table 1). Differences in manganese concentrations between groups in samples and protein pellets were similar to those for copper, except for the H150-120 group, which had a higher concentration than the S150-120 group (*p* < 0.05) (Table 1).

### 2.3. Expression Analysis of Protein Spot

Expression analysis revealed a total of 40 differentially expressed spots (“minimum fold change” of 1.3, *p* < 0.05) between groups. The proteins identified in the spots had pIs between 4.80 and 9.07 and molecular masses between 40 and 209 kDa. In 9 spots, no proteins were identified, or they had proteins with a very low score disqualifying them from the study. Table 2 shows 31 spots characterized by their respective proteins and differences in relative abundance between groups. The number of spots characterized by significant expression in each group comparison was 8 spots at S150-120 versus S15-80; 14 spots at H15-80 versus S15-80; 10 spots in H150-120 versus S150-120; and 5 spots at H150-120 versus H15-80. Supplementary Table S1 shows the complete information regarding expression analysis (score, gene and theoretical pI/MM).

The results of the analysis of genetic ontology terms (GO) can be seen in Supplementary Figure S1, showing the biological processes, molecular functions and cellular components in which the proteins were associated.

**Figure 2:**
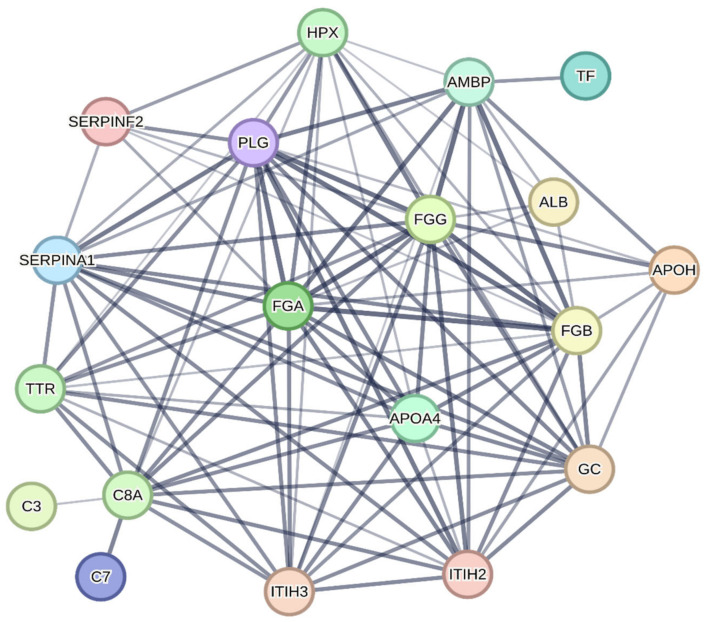
Protein-protein interaction network of proteins identified in differentially expressed protein spots on polyacrylamide gels from plasma samples from broiler chickens supplemented with different levels and sources of copper and manganese. Each node (colored spheres) represents a protein with the name of the respective centralized gene. The lines connecting each node indicate evidence of interaction between the proteins, and the thickness of the lines indicates the confidence level of the interaction (the thicker the line, the greater the confidence level). ALB: Albumin; AMBP: Protein AMBP; APOA4: Apolipoprotein A4; APOH: Beta-2-glycoprotein 1; C3: Complement C3 precursor; C7: Complement component 7; C8A: Complement C8 alpha chain; GC: Vitamin D-binding protein; FGA: Fibrinogen alpha chain; FGB: Fibrinogen beta chain; FGG: Fibrinogen gamma chain; HPX: Hemopexin; ITIH2: Interalpha-trypsin inhibitor heavy chain 2; ITIH3: Interalpha-trypsin inhibitor heavy chain 3; PLG: Plasminogen; SERPINA1: Serpin peptidase inhibitor_ clade A (alpha-1 antiproteinase_ antitrypsin)_ member 1; SERPINF2: Serpin family F member 2; TF: Ovotransferrin; TTR: Transthyretin.

The protein-protein interaction network is shown in Figure 2. According to the criteria adopted during the analysis, evidence of interaction was found in 19 proteins (ALB, AMBP, APOA4, APOH, C3, C7, C8A, GC, FGA, FGB, FGG, HPX, ITIH2, ITIH3, PLG, SERPI-NA1, SERPINF2, TF, and TTR).

**Figure 3:**
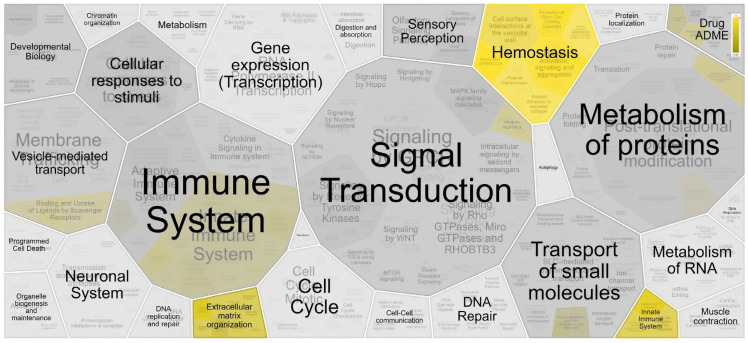
Hierarchical view of the analysis of the pathway of the proteins identified in the differentially expressed spots, highlighting the pathways of hemostasis, extracellular matrix organization and the innate immune system. Pathway analysis was performed using the Reactome database tool.

Reactome pathway analysis resulted in 36 significantly enriched pathways (FDR < 0.05), as shown in Supplementary Table S2. Figure 3 shows the hierarchical view of the pathway analysis based on the overrepresentation of the *p* value, demonstrating the involvement of proteins in hemostasis pathways, extracellular matrix organization and the innate immune system.

## 3. Discussion

### 3.1. Cu and Mn Concentrations in Plasma and Protein Pellet Samples

Groups supplemented with higher levels of copper and manganese had higher concentrations of these minerals in plasma samples and protein pellets compared to groups that received levels as needed, regardless of source. Similar results have been reported in other studies suggesting that the concentration of microminerals increases in different tissues in response to higher levels of inclusion in diets [12,15,16,18]. While this elevation in tissues may seem beneficial in meeting the physiological demands of birds, it also poses potential risks that need to be considered. The main risk is the possibility of mineral toxicity, especially when levels exceed tolerable limits. This can lead to organ damage, metabolic imbalances, interference with the absorption of other essential nutrients, inflammatory responses and oxidative stress [19,20,21,22]. Consequently, all these effects, in addition to damaging the health of the birds, can affect the final quality of the meat. Therefore, more studies need to be carried out to determine the physiological limits in broilers and how high levels of these minerals can compromise meat quality attributes.

Regarding the sources, it was observed that there was no difference between sulfate and Cu hydroxychloride when supplemented at the highest level (150 mg kg^−1^ Cu). Interestingly, the groups supplemented with 15 mg kg^−1^ Cu from the hydroxychloride source showed higher Cu concentrations in the protein samples and pellets compared to sulfates, demonstrating that Cu sulfate was less efficient when the minimum required concentration was used. On the other hand, Mn concentrations both in the samples and in the pellets were higher in the groups supplemented with hydroxychlorides regardless of the supplementary level, indicating a greater capacity of Mn hydroxychloride to make the mineral available for absorption and incorporation into proteins compared to sulfates. This may be because, unlike sulfates, hydroxychlorides have a higher proportion of covalent bonds, which allows for a slower release of minerals during digestion and provides better stability in the gastrointestinal tract. In addition, compared to sulfates, hydroxychlorides also have lower hygroscopicity and water solubility, which makes them less susceptible to reactions with other nutrients in the diet, and consequently avoids antagonistic interactions that can impair the absorption process [9,10,11]. All these factors seem to contribute to the greater mineral bioavailability presented by hydroxychloride sources. However, further studies should be carried out to better understand the mechanisms involved, considering not only the chemical form of the minerals but also the interactions that occur before and after absorption, as well as the composition of the diet and the different physiological conditions of the birds.

### 3.2. Regulation of Plasma Proteins

In the present study, the use of different sources and levels of Cu and Mn promoted changes in the level of expression of plasma proteins associated with multiple processes and biological functions. Functional analysis indicated the involvement of proteins in relevant processes such as biological regulation, stress response and immune system processes. Similarly, pathway analysis highlighted hemostasis, innate immune system and extracellular matrix organization pathways for the set of identified proteins. Most of the proteins showed evidence of interactions among themselves, demonstrating the complexity of the response to the studied groups. From the analysis of the GO terms, eight proteins were associated with the function of metal binding (albumin, apolipoprotein AIV, pentraxin, fibrinogen alpha chain, fibrinogen beta chain, fibrinogen gamma chain and pyruvate kinase), highlighting that albumin binds to different metals, including copper and manganese.

Albumin is the most abundant protein in blood plasma and plays an important role in the transport of several molecules and minerals, which makes it essential in controlling oxidative stress by preventing the free circulation of metals with pro-oxidant activities [23]. The group supplemented with a lower level of Cu and Mn hydroxychloride (H15-80) showed greater expression of spots 70, 71 and 98 characterized by albumin compared to the group supplemented with sulfates (S15-80). Taking into account the metal binding properties of albumin, its upregulation in the H15-80 group possibly occurred due to the higher concentrations of Cu and Mn in the plasma and protein pellet, as shown in Table 1. On the other hand, supplementation with higher levels of Cu and Mn hydroxychloride (H150-120) decreased the expression of spots 56 and 105 in the presence of albumin and other proteins in relation to the group supplemented with the same level of sulfate (S150-120). Although there were no differences in Cu concentrations between the H150-120 and S150-120 groups, the Mn concentration was higher in the H150-120 group. Thus, the higher concentration of Mn presented by the group supplemented with hydroxychloride may have exposed the changes caused in albumin in response to higher levels of minerals, since it was suggested that in situations of excess metals, albumin adopts an attitude of “self-sacrifice” directing oxidative damage to itself to limit damage to other important molecules [24].

Another relevant observation refers to the fact that all spots in the H150-120 group showed negative regulation when compared to the S150-120 and H15-80 groups. In these spots, in addition to albumin, other proteins involved in the antioxidant capacity of plasma were also identified, such as the alpha, beta and gamma chains of fibrinogen (spots 28, 44, 104, 105 and 106) and hemopexin (spot 31). Fibrinogen, for example, was reported in previous studies to have an antioxidant function in plasma and to be highly susceptible to oxidation [25,26]. Similarly, hemopexin acts by protecting cells from free heme toxicity; however, exposure to reactive oxygen and nitrogen species causes oxidative changes in its structure, compromising its activity [27]. Although it is not clear why the H150-120 group proteins were negatively regulated, the higher mineral bioavailability presented by the hydroxychlorides may have made the effects of high levels of Cu and Mn on the plasmatic proteome more evident, suggesting an imbalance in homeostasis and possible alterations in the structure and functioning of proteins, either by the generation of free radicals or by the direct binding of metals to proteins, requiring further studies to clarify such mechanisms.

The group supplemented with higher levels of Cu and Mn sulfate (S150-120) also showed changes in protein expression when compared to the group supplemented with recommended levels (S15-80). However, the differential expression of spots with some proteins different from those found in the comparison between the H150-120 and H15-80 groups was observed, in addition to showing positive regulation in two spots, 25 and 54. In spot 25, SPIA1 (Serpin peptidase inhibitor_ clade A (alpha-1 antiproteinase_ antitrypsin)_ member 1) was identified, a protein commonly known as alpha-1-antitrypsin, belonging to the superfamily of serpin serine protease inhibitors. SPIA1 is relatively abundant in plasma, and in addition to inhibiting proteases, it is also involved in many physiological processes, such as the regulation of anti-inflammatory and immunomodulatory responses [28]. In spot 54, proteins from the inter-alpha-trypsin (IαI) inhibitor family were identified, with heavy chains 2 and 3 (ITIH2 and ITIH3) and the AMBP protein (precursor of alpha-1-microglobulin/bikunin—AMBP). Despite belonging to the family of protease inhibitors, these proteins are known to bind to hyaluronic acid, stabilizing the extracellular matrix, and have also been associated with cellular repair of tissue injuries [29,30]. Furthermore, Garantziotis et al. [31] reported the action of IαI in attenuating complement activation and tissue damage induced by complement in response to inflammation. In fact, in the present study, lower expression of the complement proteins C3, C7 and C3d (spot 48) was observed in the S150-120 group, suggesting that the higher levels of Cu and Mn sulfate may have initiated an inflammatory response leading to the induction of inter-alpha-trypsin inhibitors to control potential tissue damage.

The group supplemented with the lowest level of Cu and Mn sulfate (S15-80) showed negative regulation of almost all spots compared to the group supplemented with the same level of hydroxychlorides (H15-80), also coinciding with the lowest concentrations of minerals in samples and protein pellets. Assuming that the level of Cu and Mn supplemented in these groups was in accordance with the nutritional recommendation for broilers, with no risk of toxicity, it can be assumed that the provision of Cu and Mn from sulfates is at a suboptimal level when compared to hydroxychlorides. Although the results are insufficient to infer any negative effect, it was possible to observe that the lower absorption of minerals from sulfates induced changes in the regulation of proteins in blood plasma compared to hydroxychlorides, thus emphasizing the importance of using sources with greater mineral bioavailability to adequately meet the requirements of the birds.

## 4. Materials and Methods

The experimental procedures carried out in the present study were approved by the Committee on Ethics in the Use of Animals (CEUA) of the School of Veterinary Medicine and Animal Science of the São Paulo State University (UNESP) under protocol CEUA 0191/2018.

### 4.1. Birds, Experimental Groups and Sample Collection

For the proteomic study, plasma samples from 40 male broiler chickens of the Cobb^®^ 500 lineage were used. The birds were distributed into four experimental groups considering two sources of copper and manganese supplementation (sulfate and hydroxychloride), with levels recommended by Rostagno et al. [32] (15 and 80 mg kg^−1^ of Cu and Mn, respectively) and levels above the recommendations (150 and 120 mg kg^−1^ of Cu and Mn, respectively) (Table 3).

The birds received feed and water ad libitum through a tube feeder and nipple drinker in 2.0 m^2^ boxes lined with reused wood shaving litter. Diets based on corn and soybean meal were divided into four phases: preinitial (1–7 days); initial (8–21 days); growth (22–35 days); and final (35–42 days). The birds were warmed up to the 14th day of age using infrared lamps (250 watts), and the light program was followed according to the recommendations in the lineage manual using a timer with 20-watt lamps.

At 42 days of age, blood samples were collected via puncture of the jugular vein, followed by centrifugation at 671× *g* for 15 min in tubes with sodium heparin to obtain plasma. After plasma separation, the samples were transferred to cryotubes and immediately stored in a −80 °C freezer until proteomic analysis was performed. Prior to the proteomic assays, four pools were made from the plasma samples of 10 birds per group, resulting in the four evaluated treatments.

### 4.2. Extraction, Precipitation, and Determination of Total Protein

For protein extraction, 100 µL of pooled plasma from each group and 200 µL of ultrapure water (18.2 MΩ cm^−1^) were added to microtubes. Soon after, centrifugation was performed at 9503× *g* and 4 °C for 15 min. Subsequently, the supernatants were collected and transferred to new microtubes where 900 µL of ice-cold acetone 80% (*v*/*v*) was added, remaining under refrigeration for 2 h at 4 °C for protein precipitation. Then, the samples were centrifuged again for 10 min at 9503× *g* and 4 °C. After obtaining the protein pellets, they were solubilized in a vortex with 1000 µL of cold ethanol and centrifuged at 9503× *g* for 5 min. This procedure was performed twice. To determine the total protein concentration, a portion of the protein pellets was used following the Biuret method and using bovine serum albumin (BSA) as a standard [33]. Briefly, the pellets were solubilized in 100 μL of NaOH at 0.5 mol L^−1^, and then 50 μL aliquots of the previously solubilized pellets and 2.5 mL of Biuret reagent were added to 5 mL tubes and placed in a water bath at 32 °C for 10 min. A blank was also prepared with ultrapure water following the same procedures described above. After the water bath, the samples were kept at room temperature for 5 min and then transferred to 1 cm wide cuvettes, with absorbance readings being performed in a UV/Visible spectrophotometer at a wavelength of 545 nm.

### 4.3. Two-Dimensional Polyacrylamide Gel Electrophoresis (2D-PAGE) and Gel Analysis

For fractionation of the plasmatic proteome by 2D-PAGE, solutions containing 1.50 µg µL^−1^ of total protein were prepared from the solubilization of protein pellets in buffer containing 7 mol L^−1^ of urea, 2 mol L^−1^ of thiourea, 2% (*w*/*v*) CHAPS (3-[(3-cholaminopropyl)-dimethylammonium]-1-propane sulfonate), 0.5% (*v*/*v*) ampholytes pH ranging from 3 to 10, and 0.002% (*w*/*v*) bromophenol blue. Then, three 13 cm strips per group (containing prefabricated gel and pH gradient ampholytes from 3 to 10) were hydrated with 250 µL aliquots of the pellet solutions (corresponding to 375 µg of protein) in a specific hydration box. The strips remained for 12 h at room temperature and were covered with mineral oil to prevent sample evaporation. After 12 h, the strips were transferred to the isoelectric focusing system (IEF) EttanTMIPGphorTM 3—IEF (GE Healthcare, Uppsala, Sweden), in which the proteins were separated according to their isoelectric points (pIs), the first dimension of electrophoresis two-dimensional [34]. The running time was five hours.

At the end of the IEF procedure, the strips underwent equilibration steps for a period of 15 min with a dithiothreitol (DTT) solution under gentle agitation, followed by an iodoacetamide solution, repeating the same process. Subsequently, the strips were positioned on top of 12.5% (*v*/*v*) polyacrylamide gels prepared between glass plates (180 × 160 × 1.5 mm), and a marker was also added to the side of the strips using filter paper containing proteins with known molecular masses (β-phosphorylase: 97.0 kDa, albumin: 66.0 kDa, ovalbumin: 45.0 kDa, carbonic anhydrase: 30.0 kDa, trypsin inhibitor: 20.1 kDa and α-lactalbumin: 14.4 kDa). Then, an electrophoretic run was performed, where proteins were fractionated according to their respective molecular weights (second dimension of 2D-PAGE). For this, a voltage of 100 volts was applied during the initial 30 min and later changed to 180 volts for a period of 3 h and 40 min.

At the end of the electrophoretic run, the gels were removed from the glass plate system and immersed in a fixative solution (10% acetic acid (*v*/*v*) and 40% ethanol (*v*/*v*)) for 30 min. Then, the solution was removed, and the gels were immersed in colloidal Coomassie G-250 dye (USB, Cleveland, OH, USA) and kept under gentle agitation for a period of 72 h. Then, the dye was removed, and the gels were washed with ultrapure water until the protein spots were visible. The gels were digitalized in a specific scanner, and the images were analyzed using Image Master Platinum v.7.0 software, obtaining the number of spots, percentage of correlation (matching) between the repetitions of gels, and the isoelectric point (pI) and experimental molecular mass (MM) of each spot. Expression analysis of protein spots (upregulation and downregulation) was performed using the same software, with significant expression being considered when the spot had a minimum difference of 1.3 times in abundance (fold change) and *p* < 0.05. There were three gels per treatment and the following comparisons relevant to the study were considered: S150-120 versus S15-80; H15-80 versus S15-80; H150-120 versus S150-120; and H150-120 versus H15-80.

### 4.4. Characterization of Protein Spot

The spots that showed significant expression were extracted from the gels and sectioned into segments of approximately 1 mm^3^. Subsequently, dye removal, reduction, alkylation and tryptic digestion of proteins with trypsin solution at a concentration of 10 ng mL^−1^ were performed according to procedures described by Braga et al. [35]. Peptides were purified using C18 ZipTip columns and then concentrated in a vacuum concentrator to approximately 1 µL. Peptide sequences from tryptic digestion were characterized by liquid chromatography–tandem mass spectrometry (LC-MS/MS). The eluted peptide solutions were analyzed using the nanoAcquity UPLC system coupled to the Xevo Q-TOF G2 mass spectrometer (Waters, Manchester, UK).

### 4.5. Bioinformatics Analysis

The UniProt database was used to identify the proteins and obtain the FASTA sequences used in the ontological analysis using Blast2GO v.6.0.3 software, which classified the protein sequences into three domains: biological process, molecular function and cell component. The protein-protein interaction network was constructed using the String online database (string-db.org v.11.5) with a minimum required confidence score of 0.400 (medium confidence). The sources of interaction “experiments”, “database”, “coexpression” and “cooccurrence” were considered. Pathway analysis was performed using the Reactome pathway database tool (https://reactome.org/ accessed on 22 February 2023) with FDR < 0.05 considered significantly enriched pathways.

### 4.6. Quantification of Copper and Manganese in Protein Pellets and Plasma Samples

The copper and manganese determinations were performed using the procedures described by Cavecci et al. [36], as summarized as follows: The samples mineralization from plasma and protein pellets was performed with concentrated sulfuric acid (Baker) and hydrogen peroxide (Merck) 30% (m/m) by heating in a digester block until complete digestion (transparent extract). Subsequently, the acid extracts were increased to 5 mL in calibrated tubes, and then copper and manganese were quantified by flame atomic absorption spectrometry (FAAS) using a Shimadzu AA—6800 spectrometer. Analytical curves were constructed using copper and manganese standard solutions in 0.10 mol L^−1^ hydrochloric acid medium prepared from the dilution of Titrisol MERK standards containing 1000 mg L^−1^ of the analytical standards. For the experimental adjustments of the analyses were used also the conditions described by Cavecci et al. [36].

Copper and manganese concentrations were assessed through analysis of variance (ANOVA) using Minitab 17 software. Mean values were compared using Tukey’s test (*p* < 0.05). The data were presented as means and standard deviation.

## 5. Conclusions

The concentrations of Cu and Mn in plasma and protein pellets indicated greater mineral bioavailability in the hydroxychloride source than in sulfates. Proteomic analysis revealed changes in the expression pattern of plasma proteins involved in various pathways, providing a path for future research in the search for responsive biomarkers to different dietary concentrations of minerals. In this sense, it is suggested that future studies combine different proteomic approaches to reduce the limitations of each technique and expand the results, as well as the individual study of each metal for better clarity and understanding of the mechanisms involved in changes in the proteome and its impacts on bird health and meat quality.

## Figures and Tables

**Table 1:** Copper and manganese concentrations in plasma samples and pellets from broilers supplemented with different sources and levels of copper and manganese.

Groups	Cu Concentration	Mn Concentration
Plasma (mg L^−1^)		
S15-80	2.65 ± 0.07 ^c^	5.25 ± 0.07 ^d^
S150-120	23.40 ± 0.28 ^a^	16.85 ± 0.07 ^b^
H15-80	5.75 ± 0.07 ^b^	7.85 ± 0.07 ^c^
H150-120	20.70 ± 0.14 ^a^	20.45 ± 0.07 ^a^
Protein pellet (mg kg^−1^)		
S15-80	1.67 ± 0.12 ^c^	3.15 ± 0.09 ^d^
S150-120	37.57 ± 0.97 ^a^	25.33 ± 0.75 ^b^
H15-80	3.57 ± 0.42 ^b^	4.30 ± 0.10 ^c^
H150-120	40.13 ± 0.61 ^a^	32.70 ± 0.82 ^a^

^a–d^ Means followed by different superscript letters in the column differ by Tukey’s test (*p* < 0.05). S15-80 (15 mg kg^−1^ Cu sulfate and 80 mg kg^−1^ Mn sulfate); S150-120 (150 mg kg^−1^ Cu sulfate and 120 mg kg^−1^ Mn sulfate); H15-80 (15 mg kg^−1^ Cu hydroxychloride and 80 mg kg^−1^ Mn hydroxychloride) and H150-120 (150 mg kg^−1^ Cu hydroxychloride and 120 mg kg^−1^ Mn hydroxychloride).

**Table 2:** Proteins characterized in protein spots differentially expressed in polyacrylamide gels from pools of plasma from broilers supplemented with two sources (sulfates (S) and hydroxychlorides (H)) and two levels of Cu (15 and 50 ppm) and Mn (80 and 120 ppm).

Spot ID	Accession	Protein	“Fold-Change”			
			H15-80 versus S15-80	H150-120 versus S150-120	H150-120 versus H15-80	S150-120 versus S15-80
3	R9PXM5	Immunoglobulin-lambda-like polypeptide 1			−1.362/+1.362	
	A0A3Q2UDC8	Ig-like domain-containing protein			−1.362/+1.362	
13	O93601	Apolipoprotein AIV				−1.325/+1.325
25	E1C7T1	Serpin peptidase inhibitor_ clade A (alpha-1 antiproteinase_ antitrypsin)_ member 1				+1.424/−1.424
	E1BV78	Fibrinogen gamma chain				+1.424/−1.424
26	F1P4V1	Fibrinogen alpha chain	+2.438/−2.438			
28	F1NUL9	Fibrinogen beta chain		−1.478/+1.478		
31	Q90WR3	Hemopexin (Fragment)		−1.740/+1.740		
38	F1NWX6	Plasminogen		−1.625/+1.625	−1.647/+1.647	
	Q7LZF3	Plasmin		−1.625/+1.625	−1.647/+1.647	
44	F1NUL9	Fibrinogen beta chain	+1.919/−1.919		−1.339/+1.339	−1.753/+1.753
	Q9W6F5	Vitamin D-binding protein	+1.919/−1.919		−1.339/+1.339	−1.753/+1.753
48	Q90633	Complement C3				−1.328/+1.328
	F1DQG4	Complement component 7				−1.328/+1.328
	A6N9E0	Complement component 3d (Fragment)				−1.328/+1.328
50	A0A3Q2U3V9	Beta-microseminoprotein-like				−1.626/+1.626
	Q7LZS1	12K serum protein_ beta-2-m cross-reactive (Fragment)				−1.626/+1.626
54	A0A1D5NXA6	Inter-alpha-trypsin inhibitor heavy chain 3				+1.373/−1.373
	B3VE14	Inter-alpha inhibitor heavy chain 2				+1.373/−1.373
	A0A1D5PU00	Protein AMBP				+1.373/−1.373
56	A0A3Q2UFG5	Ig-like domain-containing protein		−1.426/+1.426		
	A2N881	VH1 protein		−1.426/+1.426		
	A0A1D5NW68	Albumin		−1.426/+1.426		
	F1NJU5	Complement C8 alpha chain		−1.426/+1.426		
59	A0A1I7Q422	Transthyretin			−1.349/+1.349	
64	A0A1D5PW77	C-reactive protein_ pentraxin-related	+2.030/−2.030			
	Q2EJU6	Pentraxin	+2.030/−2.030			
67	F1NW43	Pyruvate kinase	+1.802/−1.802			
	F1NUL9	Fibrinogen beta chain	+1.802/−1.802			
68	F1NUL9	Fibrinogen beta chain	+2.262/−2.262			
	F1NW43	Pyruvate kinase	+2.262/−2.262			
	A0A1D5PNU2	Beta-2-glycoprotein 1	+2.262/−2.262			
	F1P4V1	Fibrinogen alpha chain	+2.262/−2.262			
70	A0A3Q2UFG5	Ig-like domain-containing protein	+2.414/−2.414			
	A0A1D5NW68	Albumin	+2.414/−2.414			
	A2N881	VH1 protein	+2.414/−2.414			
	F1ND07	Proteasome activator subunit 4	+2.414/−2.414			
71	A0A3Q2UFG5	Ig-like domain-containing protein	+5.128/−5.128			
	A0A1D5NW68	Albumin	+5.128/−5.128			
	A2N881	VH1 protein	+5.128/−5.128			
72	Q4ADJ7	Ovotransferrin	−1.394/+1.394			
73	A0A3Q2UFG5	Ig-like domain-containing protein		−1.439/+1.439		
	A2N881	VH1 protein		−1.439/+1.439		
75	F1NWX6	Plasminogen	+1.741/−1.741			
	Q7LZF3	Plasmin	+1.741/−1.741			
79	A0A3Q2U3V9	Beta-microseminoprotein-like	+1.382/−1.382			
	Q7LZS1	12K serum protein_ beta-2-m cross-reactive (Fragment)	+1.382/−1.382			
98	A0A1D5NW68	Albumin	+2.293/−2.293			
100	A0A1L1RIW5	Keratin 8		−2.011/+2.011		
104	A0A1D5PNU2	Beta-2-glycoprotein 1		−1.507/+1.507		
	F1NUL9	Fibrinogen beta chain		−1.507/+1.507		
	F1P4V1	Fibrinogen alpha chain		−1.507/+1.507		
	F1NW43	Pyruvate kinase		−1.507/+1.507		
	A0A1L1RIW5	Keratin 8		−1.507/+1.507		
105	E1BV78	Fibrinogen gamma chain		−1.909/+1.909		
	A0A1D5NW68	Albumin		−1.909/+1.909		
	F1ND07	Proteasome activator subunit 4		−1.909/+1.909		
106	Q98TD1	PIT 54			−1.308/+1.308	
	A0A3Q2UF48	Peptidase S1 domain-containing protein			−1.308/+1.308	
	E1BV78	Fibrinogen gamma chain			−1.308/+1.308	
	A0A1D5NW68	Albumin			−1.308/+1.308	
107	A0A3Q2UFG5	Ig-like domain-containing protein		−2.109/+2.109		
108	F1NAR5	Serpin family F member 2		−1.703/+1.703		
	A0A1L1RIW5	Keratin 8		−1.703/+1.703		
111	F1NK40	Alpha-2-macroglobulin-like 4				−1.425/+1.425
	E1BV78	Fibrinogen gamma chain				−1.425/+1.425
	H1AC38	A2M_recep domain-containing protein (Fragment)				−1.425/+1.425
112	Q90WR3	Hemopexin (Fragment)	−2.550/+2.550			
	A0A1D5PEU7	Vanin 1	−2.550/+2.550			
	Q5ZHM4	CN hydrolase domain-containing protein	−2.550/+2.550			

S15-80 (15 mg kg^−1^ Cu sulfate and 80 mg kg^−1^ Mn sulfate), S150-120 (150 mg kg^−1^ Cu sulfate and 120 mg kg^−1^ Mn sulfate), H15-80 (15 mg kg^−1^ Cu hydroxychloride and 80 mg kg^−1^ Mn hydroxychloride) and H150-120 (150 mg kg^−1^ Cu hydroxychloride and 120 mg kg^−1^ Mn hydroxychloride).

**Table 3:** Description of experimental groups.

Experimental Groups		Cu (mg kg^−1^)	Mn (mg kg^−1^)
Sulfates	S15-80	15	80
	S150-120	150	120
Hydroxychlorides	H15-80	15	80
	H150-120	150	120

## Data Availability

The datasets generated and analyzed during the current study are available in the UNESP Institutional Repository (http://hdl.handle.net/11449/242914).

## Supplementary Materials

The following supporting information can be downloaded at: https://www.mdpi.com/article/10.3390/molecules28248155/s1, Figure S1. Genetic ontology analysis of the plasma proteome of broiler chickens supplemented with different levels and sources of copper and manganese using Blast2GO. Table S1. Proteins characterized in protein spots differentially expressed in polyacrylamide gels from pools of plasma from broilers supplemented with two sources (sulfates (S) and hydroxychlorides (H)) and two levels of copper (15 and 150 mg kg^−1^) and manganese (80 and 120 mg kg^−1^). Table S2. Significantly enriched pathways (FDR<0.05) using the Reactome pathway database.
