# Supplementary material for: Proteomic Study of Broiler Plasma Supplemented with Different Levels of Copper and Manganese from Different Sources
Source: Molecules. 2023 Dec 18;28(24):8155. doi: 10.3390/molecules28248155 (PMC10745542; doi:10.3390/molecules28248155)
Supplement: Supplementary file 1 [file molecules-28-08155-s001.zip › molecules-2738588-supplementary.pdf]

SUPPLEMENTARY MATERIAL

Proteomic study of broiler plasma supplemented with different levels of copper and manganese from different sources

Renata Aparecida Martins[a*], Andrey Sávio de Almeida Assunção[a], José Cavalcante Souza Vieira[b], Leone Campos Rocha[a], Priscila Michelin Groff Urayama[a], Marília Afonso Rabelo Buzalaf[c], José Roberto Sartori[a], Pedro de Magalhães Padilha[b*]

[a]*School of Veterinary Medicine and Animal Science, São Paulo State University (UNESP), Botucatu, São Paulo, Brazil.*

[b]*Institute of Biosciences, São Paulo State University (UNESP), Botucatu, São Paulo, Brazil.*

[c]*University of São Paulo, (USP), Bauru, Brazil.*

***Corresponding author**

Renata Aparecida Martins, School of Veterinary Medicine and Animal Science, São Paulo State University (UNESP), Street Prof. Dr. Walter Mauricio Correa, w/n Botucatu, São Paulo, 18618-681, Brazil. E-mail: renata.a.martins@unesp.br

Pedro de Magalhães Padilha, Institute of Biosciences, São Paulo State University, Street Prof. Dr. Antonio Celso Wagner Zanin, 250, Botucatu, São Paulo, 18618-693, Brazil. E-mail: pedro.padilha@unesp.br

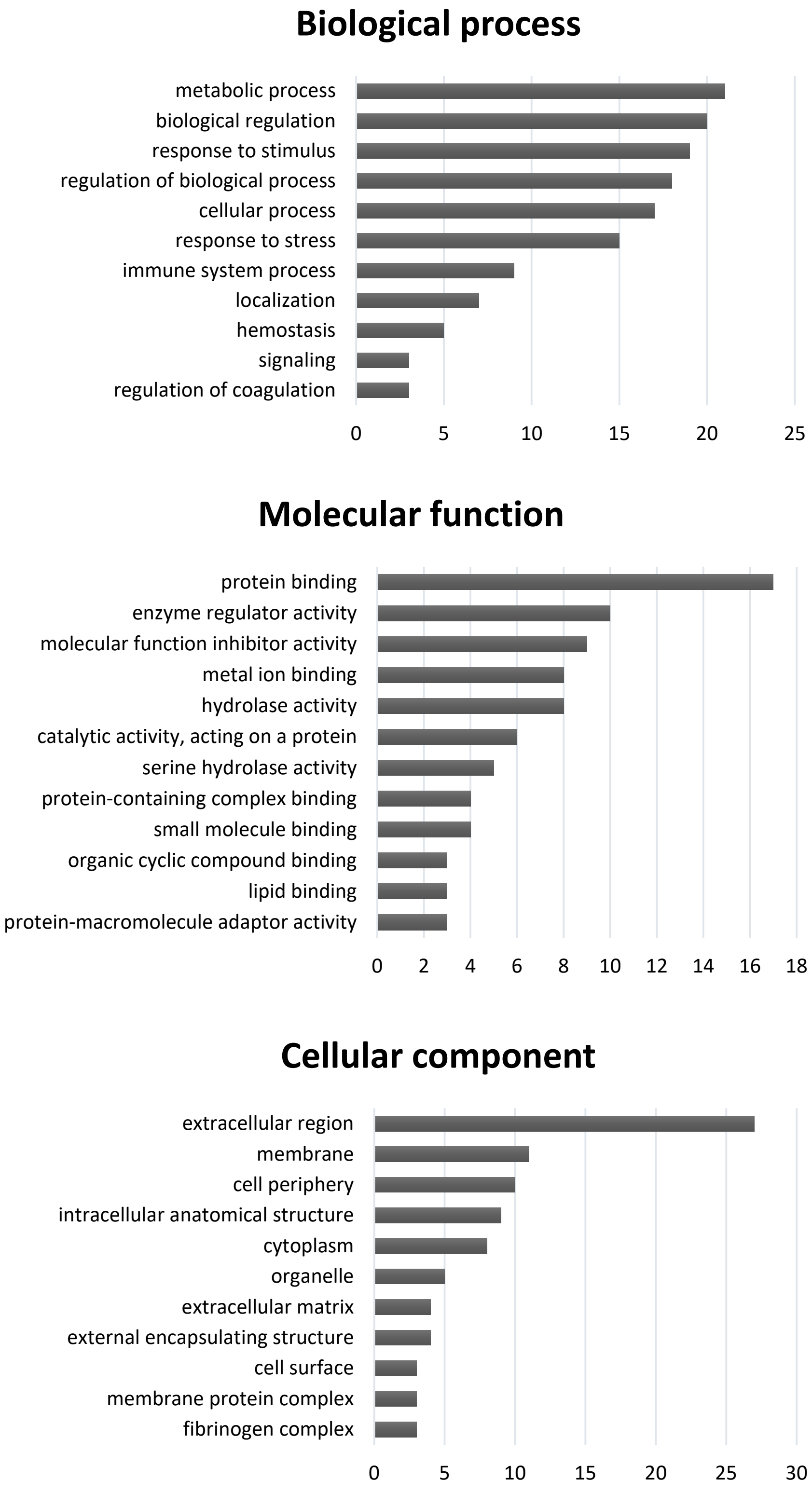

Supplementary Figure S1. Genetic ontology analysis of the plasma proteome of broiler chickens supplemented with different levels and sources of copper and manganese using Blast2GO.

Page Break

Supplementary Table S1. Proteins characterized in protein spots differentially expressed in polyacrylamide gels from pools of plasma from broilers supplemented with two sources (sulfates (S) and hydroxychlorides (H)) and two levels of copper (15 and 150 mg kg^{-1}) and manganese (80 and 120 mg kg^{-1}).

Spot ID	Acession	Gene	Protein	pI/MM (Da) Theoretical	Score	H15-80 *versus* S15-80	H150-120 *versus* S150-120	H150-120 *versus* H15-80	S150-120 *versus* S15-80
3	R9PXM5	N/A	Immunoglobulin lambda like polypeptide 1	6.09/ 11417.69	2343.459			-1.362/+1.362	
	A0A3Q2UDC8	N/A	Ig-like domain-containing protein	4.99/ 9762.55	738.1473			-1.362/+1.362	
13	O93601	apoAIV	Apolipoprotein AIV	4.80/ 40852.86	1734.531				-1.325/+1.325
25	E1C7T1	SPIA1	Serpin peptidase inhibitor_ clade A (alpha-1 antiproteinase_ antitrypsin)_ member 1	6.08/ 48714.69	290.6664				+1.424/-1.424
	E1BV78	FGG	Fibrinogen gamma chain	5.47/ 49954.83	3348.089				+1.424/-1.424
26	F1P4V1	FGA	Fibrinogen alpha chain	5.69/82392.35	2692.128	+2.438/-2.438			
28	F1NUL9	FGB	Fibrinogen beta chain	7.85/ 55319.58	2491.537		-1.478/+1.478		
31	Q90WR3	N/A	Hemopexin (Fragment)	5.92/ 29384.78	15930.77		-1.740/+1.740		
38	F1NWX6	PLG	Plasminogen	7.92/ 90764.21	1504.657		-1.625/+1.625	-1.647/+1.647	
	Q7LZF3	N/A	Plasmin	8.29/10325.29	369.8466		-1.625/+1.625	-1.647/+1.647	
44	F1NUL9	FGB	Fibrinogen beta chain	7.85/ 55319.58	1836.466	+1.919/-1.919		-1.339/+1.339	-1.753/+1.753
	Q9W6F5	VTDB	Vitamin D-binding protein	6.47/ 53686.59	1368.327	+1.919/-1.919		-1.339/+1.339	-1.753/+1.753
48	Q90633	N/A	Complement C3	6.69/ 184086.42	864.9603				-1.328/+1.328
	F1DQG4	C7	Complement component 7	5.96/ 93012.62	292.7017				-1.328/+1.328
	A6N9E0	C3d	Complement component 3d (Fragment)	9.01/ 36550.12	255.618				-1.328/+1.328
50	A0A3Q2U3V9	LOC100858647	Beta-microseminoprotein-like	8.32/ 12421.55	1290.518				-1.626/+1.626
	Q7LZS1	N/A	12K serum protein_ beta-2-m cross-reactive (Fragment)	5.01/ 3974.64	654.4376				-1.626/+1.626
54	A0A1D5NXA6	ITIH3	Inter-alpha-trypsin inhibitor heavy chain 3	5.58/ 72500.59	2022.493				+1.373/-1.373
	B3VE14	ITIH2	Inter-alpha inhibitor heavy chain 2	6.92/ 106767.87	903.7598				+1.373/-1.373

	A0A1D5PU00	AMBP	Protein AMBP	5.59/ 38457.56	878.0583				+1.373/-1.373
56	A0A3Q2UFG5	N/A	Ig-like domain-containing protein	9.07/ 10384.62	3791.357		-1.426/+1.426		
	A2N881	N/A	VH1 protein	9.10/12247.88	1697.29		-1.426/+1.426		
	A0A1D5NW68	ALB	Albumin	5.29/ 64004.39	767.5627		-1.426/+1.426		
	F1NJU5	C8A	Complement C8 alpha chain	6.11/ 67725.13	431.8983		-1.426/+1.426		
59	A0A1I7Q422	TTR	Transthyretin	4.97/ 18539.00	1194.293			-1.349/+1.349	
64	A0A1D5PW77	LOC776376	C-reactive protein_ pentraxin-related	6.31/ 34987.04	1473.075	+2.030/-2.030			
	Q2EJU6	CRP	Pentaxin	6.33/ 25660.32	260.4364	+2.030/-2.030			
67	F1NW43	PKLR	Pyruvate kinase	7.97/ 57873.81	2774.254	+1.802/-1.802			
	F1NUL9	FGB	Fibrinogen beta chain	7.85/ 55319.58	1133.725	+1.802/-1.802			
68	F1NUL9	FGB	Fibrinogen beta chain	7.85/ 55319.58	2847.544	+2.262/-2.262			
	F1NW43	PKLR	Pyruvate kinase	7.97/ 57873.81	1418.43	+2.262/-2.262			
	A0A1D5PNU2	APOH	Beta-2-glycoprotein 1	8.60/ 41093.29	361.6708	+2.262/-2.262			
	F1P4V1	FGA	Fibrinogen alpha chain	5.69/82392.35	247.5071	+2.262/-2.262			
70	A0A3Q2UFG5	N/A	Ig-like domain-containing protein	9.07/ 10384.62	4344.046	+2.414/-2.414			
	A0A1D5NW68	ALB	Albumin	5.29/ 64004.39	3554.366	+2.414/-2.414			
	A2N881	N/A	VH1 protein	9.10/12247.88	2203.052	+2.414/-2.414			
	F1ND07	PSME4	Proteasome activator subunit 4	6.67/ 209793.34	162.0611	+2.414/-2.414			
71	A0A3Q2UFG5	N/A	Ig-like domain-containing protein	9.07/ 10384.62	1259.289	+5.128/-5.128			
	A0A1D5NW68	ALB	Albumin	5.29/ 64004.39	658.7015	+5.128/-5.128			
	A2N881	N/A	VH1 protein	9.10/12247.88	886.9579	+5.128/-5.128			
72	Q4ADJ7	TFEW	Ovotransferrin	6.85/ 77831.61	12703.08	-1.394/+1.394			
73	A0A3Q2UFG5	N/A	Ig-like domain-containing protein	9.07/ 10384.62	4764.221		-1.439/+1.439		

	A2N881	N/A	VH1 protein	9.10/12247.88	1486.285		-1.439/+1.439	
75	F1NWX6	PLG	Plasminogen	7.92/ 90764.21	1435.87	+1.741/-1.741		
	Q7LZF3	N/A	Plasmin	8.29/10325.29	580.665	+1.741/-1.741		
79	A0A3Q2U3V9	LOC100858647	Beta-microseminoprotein-like	8.32/ 12421.55	689.0574	+1.382/-1.382		
	Q7LZS1	N/A	12K serum protein_ beta-2-m cross-reactive (Fragment)	5.01/ 3974.64	515.8345	+1.382/-1.382		
98	A0A1D5NW68	ALB	Albumin	5.29/ 64004.39	6786.945	+2.293/-2.293		
100	A0A1L1RIW5	KRT8	Keratin 8	5.79/ 60513.22	151.5625		-2.011/+2.011	
104	A0A1D5PNU2	APOH	Beta-2-glycoprotein 1	8.60/ 41093.29	3404.912		-1.507/+1.507	
	F1NUL9	FGB	Fibrinogen beta chain	7.85/ 55319.58	3101.555		-1.507/+1.507	
	F1P4V1	FGA	Fibrinogen alpha chain	5.69/82392.35	2380.485		-1.507/+1.507	
	F1NW43	PKLR	Pyruvate kinase	7.97/ 57873.81	363.0029		-1.507/+1.507	
	A0A1L1RIW5	KRT8	Keratin 8	5.79/ 60513.22	347.331		-1.507/+1.507	
105	E1BV78	FGG	Fibrinogen gamma chain	5.47/ 49954.83	19501.08		-1.909/+1.909	
	A0A1D5NW68	ALB	Albumin	5.29/ 64004.39	1006.718		-1.909/+1.909	
	F1ND07	PSME4	Proteasome activator subunit 4	6.67/ 209793.34	171.9035		-1.909/+1.909	
106	Q98TD1	PIT 54	PIT 54	4.61/50821.51	9397.017			-1.308/+1.308
	A0A3Q2UF48		Peptidase S1 domain-containing protein	6.30/20380.13	862.9152			-1.308/+1.308
	E1BV78	FGG	Fibrinogen gamma chain	5.47/ 49954.83	315.1335			-1.308/+1.308
	A0A1D5NW68	ALB	Albumin	5.29/ 64004.39	124.195			-1.308/+1.308
107	A0A3Q2UFG5	N/A	Ig-like domain-containing protein	9.07/ 10384.62	989.2745		-2.109/+2.109	
108	F1NAR5	SERPINF2	Serpin family F member 2	4.84/ 56973.56	934.6506		-1.703/+1.703	
	A0A1L1RIW5	KRT8	Keratin 8	5.79/ 60513.22	284.1509		-1.703/+1.703	

111	F1NK40	A2ML4	Alpha-2-macroglobulin-like 4	5.98/163338.74	243.638				-1.425/+1.425
	E1BV78	FGG	Fibrinogen gamma chain	5.47/ 49954.83	203.0988				-1.425/+1.425
	H1AC38	N/A	A2M_recep domain-containing protein (Fragment)	6.15/ 30756.72	114.814				-1.425/+1.425
112	Q90WR3	N/A	Hemopexin (Fragment)	5.92/ 29384.78	1525.618	-2.550/+2.550			
	A0A1D5PEU 7	VNN1	Vanin 1	5.17/ 54664.46	613.8879	-2.550/+2.550			
	Q5ZHM4	RCJMB04	CN hydrolase domain-containing protein	6.33/ 55399.28	595.0783	-2.550/+2.550			

S15-80 (15 mg kg^{-1} Cu sulfate and 80 mg kg^{-1} Mn sulfate), S150-120 (150 mg kg-1 Cu sulfate and 120 mg kg^{-1} Mn sulfate), H15-80 (15 mg kg^{-1} Cu hydroxychloride and 80 mg kg^{-1} Mn hydroxychloride) and H150-120 (150 mg kg^{-1} Cu hydroxychloride and 120 mg kg^{-1} Mn hydroxychloride)

Supplementary Table S2. Significantly enriched pathways (FDR<0.05) using the Reactome pathway database.

Pathway identifier	Pathway name	FDR	Gene
R-GGA-114608	Platelet degranulation	7.93E-07	FGA; FGG; FGB; PLG; APOH; ALB; SERPINF2; SPIA1; ITIH3
R-GGA-76005	Response to elevated platelet cytosolic Ca2+	7.93E-07	FGA; FGG; FGB; PLG; APOH; ALB; SERPINF2; SPIA1; ITIH3
R-GGA-76002	Platelet activation, signaling and aggregation	2.08E-04	FGA; FGG; FGB; PLG; APOH; ALB; SERPINF2; SPIA1; ITIH3
R-GGA-140875	Common Pathway of Fibrin Clot Formation	2.29E-04	FGA; FGG; FGB; SPIA1
R-GGA-109582	Hemostasis	5.85E-04	FGA; FGG; IGLL1; FGB; PLG; APOH; ALB; SERPINF2; SPIA1; ITIH3
R-GGA-140877	Formation of Fibrin Clot (Clotting Cascade)	9.68E-04	FGA; FGG; FGB; SPIA1
R-GGA-372708	p130Cas linkage to MAPK signaling for integrins	9.75E-04	FGA; FGG; FGB
R-GGA-354194	GRB2:SOS provides linkage to MAPK signaling for Integrins	9.75E-04	FGA; FGG; FGB
R-GGA-2132281	Regulation of complement cascades	9.75E-04	C7; C3; C8A
R-GGA-5686938	Regulation of TLR by endogenous ligand	0.00261	FGA; FGG; FGB
R-GGA-2168880	Scavenging of heme from plasma	0.00284	IGLL1; AMBP; ALB; Ig-like domain-containing protein
R-GGA-2173782	Binding and Uptake of Ligands by Scavenger Receptors	0.00374	IGLL1; AMBP; ALB; Ig-like domain-containing protein
R-GGA-75205	Dissolution of Fibrin Clot	0.00374	PLG; SERPINF2
R-GGA-2132285	Complement Cascade	0.00374	C7; C3; C8A
R-GGA-5674135	MAP2K and MAPK activation	0.00456	FGA; FGG; FGB
R-GGA-2132267	Formation of membrane attack complex (MAC)	0.00459	C7; C8A
R-GGA-166658	Complement cascade	0.00460	IGLL1; C8A; CRP; Ig-like domain-containing protein
R-GGA-354192	Integrin signaling	0.00737	FGA; FGG; FGB
R-GGA-216083	Integrin cell surface interactions	0.00737	FGA; FGG; FGB
R-GGA-173623	Classical antibody-mediated complement activation	0.00737	IGLL1; CRP; Ig-like domain-containing protein
R-GGA-8957275	Post-translational protein phosphorylation	0.00737	FGA; FGG; ALB; ITIH2
R-GGA-381426	Regulation of Insulin-like Growth Factor (IGF) transport and uptake by Insulin-like Growth Factor Binding Proteins (IGFBPs)	0.00895	FGA; FGG; ALB; ITIH2
R-GGA-9757110	Prednisone ADME	0.00928	ALB; SPIA1
R-GGA-166786	Creation of C4 and C2 activators	0.00929	IGLL1; CRP; Ig-like domain-containing protein
R-GGA-76009	Platelet Aggregation (Plug Formation)	0.01037	FGA; FGG; FGB
R-GGA-166663	Initial triggering of complement	0.01074	IGLL1; CRP; Ig-like domain-containing protein
R-GGA-8963899	Plasma lipoprotein remodeling	0.02497	PLG; ALB
R-GGA-2132287	Creation of alternative pathway C3 convertase	0.02497	C3
R-GGA-2132293	Formation of fluid-phase convertase C3	0.02497	C3

R-GGA-2132273	Formation of membrane-bound convertase C3	0.02497	C3
R-GGA-2422406	Innate Immune System	0.02879	C7; C3; C8A
R-GGA-2173345	Anaphylatoxins initiate inflammatory responses	0.03735	C3
R-GGA-9793528	Ciprofloxacin ADME	0.03735	ALB
R-GGA-168249	Innate Immune System	0.03915	FGA; FGG; FGB; IGLL1; TTR; C8A; SPIA1; CRP; Ig-like domain-containing protein
R-GGA-1474244	Extracellular matrix organization	0.04035	FGA; FGG; FGB; PLG
R-GGA-2029481	FCGR activation	0.04372	IGLL1; Ig-like domain-containing protein
